# Antitoxic Serums1Read before the Alumni Association of the Medical Department of the University of Buffalo, April 26, 1898.

**Published:** 1898-07

**Authors:** E. M. Houghton

**Affiliations:** Lecturer on experimental pharmacology, Detroit College of Medicine


### BUFFALO MEDICAL JOURNAL.

VOL. XXXVII. JULY, 1898. NO. 12

### Original Communications.

### ANTITOXIC SERUMS.[1]

BY E. M. HOUGHTON, M. D.,
Lecturer on experimental pharmacology, Detroit College of Medicine.

LIFE is, indeed, a battle. In the whole realm of living matter since its beginning an ever-continuous war has been waged. The battle is fiercely raging all about us and the fight for supremacy will continue until life ceases to exist. Since we now know that all living organisms are made up of isolated cells, or aggregations of single cells, we may well observe the phenomenon of life as presented by the unicellular organisms and trace out the general relations of the various kinds of cells to one another, and then we shall have a clearer understanding of the methods of attack and defense as presented by the ordinary animals and man toward the single-cell microscopical plant, the disease-producing or pathogenic bacteria. When we realise that at least one-third of the human family die of diseases known to be of bacterial origin we can understand with what a relentless foe we have to contend. However, if germs are the greatest enemies of mankind they are also his greatest friends, since the transformation of organic substances into inorganic constituents, of which they are composed, is dependent upon the ceaseless energy of these lowest forms of life. Without them the world would be uninhabitable—a vast sepulcher filled with the unchanged dead. For ages many of the facts of immunity have been known and studied by the medical profession, but these facts and the therapeutic application of remedies were often fallacious. From the invention of the microscope dates the dawn of rational medicine. In the course of time bacteria were discovered. Now their life history has been studied

1. Read before the Alumni Association of the Medical Department of the University of Buffalo, April 26, 1898.

and their modes of attack partially uncovered; consequently we are now able in some instances to drive the enemy from his stronghold.

The morphology of different species of bacteria varies greatly. The pigmentary bacillus prodigiosus thrives well at ordinary temperature, while most of the pathogenic bacteria must be grown in an incubator. We are able to produce culture media for many of the disease-producing organisms, but some refuse any pabulum we can offer. Other germs, as the streptococcus pyogenes, when grown artificially fail to produce the specific disease, or produce only a modified form of it when injected into susceptible animals. Still others, as the diphtheria germ, may, by proper methods of cultivation, be kept virulent for years.

The products formed by bacteria vary greatly, depending upon the food supply and the conditions under which development takes place. Carbonic acid, alcohols and gases of the methane series are some of the intermediate products that may be formed during the disintegration of the carbohydrates by bacteria. Proteid substances are the origin of a still greater variety of combinations before being returned in the form of nitrites, acids and other inorganic substances to support vegetable life. These intermediate products of carbohydrate or proteid disintegration may be merely decomposition products, or they may be the result of synthetic processes of bacteria. Many of them are of the greatest practical importance to mankind and to the medical profession.

The ptomaines—that class of bacterial products that bear such a striking resemblance to the vegetable alkaloids—may or may not be poisonous. Breiger and others have isolated many of them in a pure state and determined their formulæ.

More poisonous are the proteid bodies, sometimes called toxalbumins, some of which behave chemically like albumins, others like globulins, while still others resemble ferments most closely. These belong to the same group of poisons as the deadly ricin from the castor-oil bean, similar bodies from other plants, and the poisons isolated from the venom of snakes.

The most deadly weapons of bacteria are not their ptomaines, or toxalbumins. They produce other substances, which fail to give the biuret test or to respond in a characteristic manner to the alkaloidal reagents, but which are many times more poisonous than either ptomaines or toxalbumins. For want of a better name these ultra-poisonons products are called toxins. They have not been isolated in a pure state, consequently we know neither their composition nor the minimum lethal dose, but from the fact that a grain of impure tetanus toxin is sufficient to kill several hundred men we must conclude that the toxicity of these products is without a parallel in the whole realm of science.

In the production of antitoxic serums it is necessary first to obtain the specific bacterial products in solution for injection into the animals that are to be immunised. Owing to the fact that some bacteria, as the tubercle bacillus, bacillus typhi abdominalis and the like, contain in their enveloping membrane a wax-like material, which prevents the substances formed in the interior of the cell from being dissolved in the culture-medium in which it is grown, it has been found necessary (as shown by Koch, Buchner and others) to devise mechanical means for destroying this envelope, in order to obtain the specific products of the microörganism in solution. Other bacteria, as the diphtheria germ, do not have this impervious membrane, and their specific products readily pass into solution in the environing culture medium in which they are grown. This flask contained two weeks ago a straw-colored, sterile, transparent beef bouillon. It was planted with pure cultures of the Klebs-Löffler diphtheria germ, and placed in the incubator. The fluid has become turbid. This turbidity is mainly due to the countless millions of dead and living diphtheria cells. I have filtered a portion of the culture through this unglazed porcelain filter, which has meshes so fine that all foreign material not in solution is kept back. Yet the specific poisons of the germ are contained in the transparent filtrate which has passed through the filter and which has the exact appearance of the original bouillon.

It seems almost incredible, yet it is true, that a horse weighing over 1,200 pounds, which was under my care, died with all the symptoms of diphtheria paralysis about ten days after being injected with two minims of this innocent-looking fluid. When we remember that at least 98 per cent. of the two minims injected was nothing but water, and that a large proportion of the other possible 2 per cent. was composed of salts and peptone, we can form some conception of the potency of the products formed by the dreaded diphtheria germ. Another and smaller animal, which had been under treatment for over two years, standing in an adjoining stall, was given over ten thousand times the above quantity of the same poisonous fluid without showing any marked reaction to it.

The mass of facts already determined by the army of busy workers regarding natural and acquired immunity is so great, and the evidence is so conflicting that it is wellnigh impossible to draw definite conclusions. I shall attempt to speak of a very few of the more important points that have been studied. Animal experiments have already shown that different species of bacteria react very differently to mineral and other poisons. Thus the bacillus of chicken cholera is killed by 1-25,000 mercuric chloride, while the pneumonia bacillus of Friedlander may survive after being exposed to 1-2,000 solution. Haffkin has shown that the aqueous humor of the rabbit's eye quickly kills the bacillus typhi abdominalis, but by mixing small and gradually increasing quantities of this fluid with the culture medium in which the germs are grown the organisms become so thoroughly immunised against its toxic action, that more luxuriant growth occurs on the mixed culture medium than upon that originally employed. Certain plasmodia can be gradually accustomed to the presence of such substances as sugar, arsenic and the like, which were at first fatal to them. It appears that the natural or acquired immunity of these lower forms of life is dependent upon some peculiar property or properties possessed by the individual cells. The question naturally suggests itself, What is the explanation of the immunity possessed or acquired by higher organisms? Why should the cow be very susceptible to tuberculosis, while the horse rarely has the disease? Why can the Indian snake-charmer be bitten by the cobra without harm, while to ordinary people such bites would mean certain death? Why should one man die from smallpox while another, equally exposed, is not attacked, and still a third has the disease, but after recovery will not contract the disease again? These and similar queries have been perplexing the minds of the profession and laymen since the dawn of reason. Our attempts to answer have often been worse than confessions of ignorance until experimental methods were applied to the knotty problems.

The exhaustion theory, as the hypothesis of Pasteur was called, almost as soon as announced was disputed by Chauveau, who believed that bacteria ceased to grow, and finally died in culture flasks, and that men and animals recovered from bacterial infection when the excretory products of the disease germs had accumulated in sufficient quantities to destroy the invading microbes in the same manner as higher plants and animals are destroyed by their own excretia. Immunity, it was explained, was due to the very slow elimination of these bacterial products from the organism of the subject. This theory was accepted by Koch to explain the curative results of tuberculin. Klebs and others endeavored to find similar remedies for other of the infectious diseases after this method, but without success. Today we repudiate the explanation, although the facts hold true.

Sternberg, Metchnikoff and others have made many experiments which prove that certain of the white cells of the blood serve as police to prevent the entrance of microörganisms, and to destroy those that have already gained access to the animal body. Certain it is that the leucocytes do hasten to the point of infection and pick up many of the bacteria present, but these bacteria may have been first killed by other agencies. Metchnikoff has shown that the phagocytes may be educated, by means of the subcutaneous inoculation of their host with attenuated cultures, to great activity in apprehending the entrance of certain bacteria. But the theory of phagocytosis is only a partial explanation of the phenomenon. Much of the experimental work goes to show that other elements than the leucocytes defend the organism against disease. Fodor ten years ago proved that anthrax germs are destroyed by freshly drawn blood. Nuttall confirmed and extended Fodor's work, while Behring and Neissen found that the bactericidal properties of the blood of different animals vary greatly; thus the bacillus of typhoid fever was killed, while the streptococcus pyogenes was but slightly affected, by the freshly drawn blood of rabbits. Vaughan, McClintock and others have shown that the germicidal properties of blood probably depend upon nuclein which may come from the white cells of the blood. Many other facts of importance have been worked out, but no one has yet determined the chemical or physical properties of the extremely active substance contained in the blood of immune animals which are called antitoxin. Briefly, the method of conferring upon horses and obtaining the life-giving serum may be described as follows:

The greatest care should be exercised in selecting the horses that are to be immunised. Only young, healthy animals which have been carefully inspected by a competent veterinarian, tested with tuberculin and mallein and kept under observation for several days afterwards, should be accepted.

Many methods have been employed, at the suggestion of different investigators, for producing immunity in these animals. Behring and Roux both use practically the same method—the one employed generally at the present time. Small initial subcutaneous injections of the bouillon containing toxin are made, 1-10 c.c., diluted with sterile physiologic saline solution to 1 c.c., is quite sufficient for the first injection. The degree of reaction is very variable in different horses. Some will show almost no local or general effects, while others in apparently as good condition are rapidly prostrated with even the smallest injections. The temperature may be five or even six degrees above normal and marked local swelling occur. In the writer's experience if the temperature rises more than 4.5 degrees the horse will surely die, either from the primary injection or subsequently if treated further, unless antitoxin is administered. This fact suggested to me the possibility of producing immunity in such animals by combating the marked toxic symptoms with injections of antitoxin, the value of which method has been fully confirmed by other investigators.

The great difference in reaction to the toxin on the part of different horses has been shown by Bolton to be probably due to the fact that the blood serum of some unimmunised horses contains antitoxic substances. Among other animals a similar variation in resistance to other poisons is observed. The horses that show the greatest reaction to the poison are the ones that produce the most potent serum. Indeed this vial contains serum which, as obtained directly from the blood of such an animal, possesses the remarkable potency of 2,000 units to the c.c., or 125 units to one drop. I shall have occasion to refer to this later.

The intravenous method of injecting the toxin has been employed quite extensively. General disturbance, contrary to what might be expected, is much slighter when the toxin is introduced directly into the circulation than when injected subcutaneously. Klein found that by beginning with a small injection of attenuated culture of diphtheria germs and gradually increasing the quantity and virulency of the germs at the subsequent injections, the animal is able to take large quantities of the most virulent cultures in three or four weeks' time. This method of producing immunity is quicker and less dangerous to the horses than the method of Behring, but it is impossible thereby to produce a very strong antitoxic serum even after long continued treatment. However, it may possess greater bactericidal properties than a serum obtained by injecting the toxins. This seems hardly probable since Wassermann has shown that if an animal be immunised with virulent cultures of bacillus pyocyaneus its blood will acquire strong bactericidal but weak antitoxic properties, while an animal immunised with the toxin instead of with the germs yielded a serum which possesses both germicidal and antitoxic properties in a marked degree. By all these methods strong immunity can be produced. It may be that a combination of methods will be found ultimately to be more satisfactory than any one method alone. Horses, as human beings, have their own individual characteristics, that must be taken into consideration in order that the best results may be produced. Occasionally an animal will, while under treatment, show all the typical symptoms of post-diphtheric paralysis, even regurgitate its food. Such cases recover very slowly or may suddenly die of heart failure, as do many human patients. Exercise, sunshine, fresh air and careful attention to the food, in fact, the best hygienic conditions should prevail. If such attention is given, the horses may gain in flesh.

The amount of toxin and the length of treatment necessary to immunise animals so that they will produce antitoxins of a given strength vary within wide limits. Some horses will produce a strong antitoxin for a time and then, notwithstanding the fact that we can inject very large and increasing quantities of toxin, the serum will become much weaker; or, on the other hand, as noted by Behring, an animal may become more susceptible to the toxin after a time, but still yield a very strong serum.

After the horses have been treated for a considerable time and are able to resist large amounts of the toxins, test quantities of blood are drawn from the jugular vein, observing the most careful aseptic and antiseptic precautions, into sterilised flasks or tubes and placed into the refrigerator. Some hours later, from the contraction of the clot, the serum is squeezed out and appears at the top of the tube. This serum contains the antitoxin. At first there is a very thick buffy coat, but it soon largely goes into solution, the leucocytes presumably furnishing a considerable portion of the protecting substance. This serum is drawn from the flasks or tubes with a sterilised siphon into a sterilised container, some preservative, trikresol, camphor, carbolic acid and the like, added, allowed to stand some time and is then passed through sterilised filters into sterilised containers. All the work of handling the serum should be done in a sterilised room by a person skilled in the technique of asepsis.

The therapeutic value of the serum must now be determined before it is finally filled into suitable containers for marketing. Several methods for standardising the serum have been proposed, but that of Behring and Ehrlich has been most generally accepted in this country, which is substantially as follows:

Half-grown guinea-pigs are the animals employed. Ten times the fatal dose of diphtheria toxin is mixed with variable quantities of the serum containing the antitoxin, then injected subcutaneously into the animals. To illustrate: Suppose we find that 5 milligrammes of a given toxin is the fatal dose for a half-grown guinea-pig (250 to 300 gms.); in testing, 50 milligrammes of the toxin are measured out, mixed with, say, .05 1-20 milligramme of the serum containing the antitoxin and injected into pig No. 1. Pig No. 2 receives a like amount of the toxin mixed with .04 1-25 milligrammes of the serum. After a few days we find that pig No. 1 is well and healthy; in fact, has never been sick, while pig No. 2 died in a short time. We would say that 1 milligramme of the serum, 1-20,000 c.c., or .00005, is sufficient to protect a guinea-pig against 50 milligrammes of toxin and that each cubic centimeter of the serum contains 2,000 antitoxic units—an antitoxic unit being ten times the quantity of serum necessary to protect a medium-sized pig against ten times the fatal dose of diphtheria toxin. The serum in this bottle, to which I have already called your attention, showed a strength of 2,000 units. This means that we could inject into each of 200,000 guinea-pigs a fatal dose of this diphtheria poison, mixed with 1 c.c. of sterile saline solution taken from a cask holding about fifty gallons, into which had been mixed 1 c c., or 15 drops of this serum. Instead of having several carloads of dead animals, as would have been the result had no serum been mixed with the toxin, the pigs would nearly or quite all remain well and happy. It seems indeed wellnigh a miracle that specific medicine should have arrived at such a stage of perfection. This serum is about twenty times as strong as those on the market three years ago.

The assaying of antitoxin is very difficult, owing to the fact that a toxin which has been kept for some time loses some of its killing properties, but will still neutralise approximately as much antitoxin as when fresh. Indeed, recently two very experienced bacteriologists, to whom I had given each a sample of serum from the same bottle for assay, reported that the serum contained 250 and 700 units respectively.

Finally, the completed and tested antitoxin is filled into sterilised containers and is ready for distribution. Some firms prefer to put up several grades of serum, using containers holding from 1 to 20 c.c. of fluid. Others believe it is better to keep the average dose as small as possible and list the number of units, irrespective of the amount of fluid. On the table will be found some of the various containers, unlabled.

The question is often asked, How long will serum keep? If properly prepared, it should keep indefinitely, in the sense of putrefying; but undoubtedly like other complex albuminous bodies, it begins to deteriorate or lose strength as soon as prepared. The greater the concentration, other conditions being the same, the more rapid the loss of antitoxic strength. Manufacturers generally add sufficient excess of serum so that the package will contain the number of units claimed on the label at the end of six months.

The following paragraphs in quotations are taken from a recent paper read by the author before the Michigan Academy of Science, *Journal of American Medical Association*, April 16, 1898 :

*Anti-venomous Serum.*—Sewall (1887) succeeded in immunising pigeons against rattlesnake poison by administering gradually increasing quantities of the venom.

Fraser (1895) found, from very extended experiments with venom of the cobra, rattlesnake and many other poisonous serpents, that animals immunised against one venom were immunised against the other venoms also ; that neither local nor general reaction follow inoculation with many times the fatal dose. Calmette and his coworkers in the Pasteur institute confirmed Fraser's work and have added many new facts bearing on the question. The protection of man from the bites of serpents, by artificial treatment with their poison, has been known and practiced for ages by the snake charmers of India. How well they have kept their secrets need not be told. The importance of this work can be appreciated from the fact that reports show that every year over 20,000 people die in India alone from the bites of snakes.

*Abrin and Ricin Antitoxin.*—Ehrlich succeeded (1891) in immunising and obtained a strong antitoxin by feeding larger and larger doses of the poisons ricin (from the castor bean) and abrin (jequirity bean). These antitoxins are specific in the true sense, since the one antitoxin does not protect an animal from the poisonous effects of the other. These results are of great importance from a scientific point of view, but of little practical importance to the physician.

*Antitetanic Serum* (lockjaw).—Kitasato (1889) showed that tetanus was due to a special germ, which, if injected into susceptible animals, produced typical symptoms of tetanus ; this germ could be obtained from the body of a person dying of the disease. Shortly afterward he succeeded in immunising rabbits, whose blood serum was found to contain antitoxic substances. These results were confirmed by Tizzoni and Cattani in 1891. Behring and others, in 1892, detailed a method for immunising horses and obtaining their serum, which was used experimentally on man. The same year Brieger and Ehrlich immunised goats and found that the protecting substance was present, not only in the blood of the animal, but also in its milk. Later he obtained the antitoxin in an impure form as a dry powder from the milk. Of the greatest importance to the physician was the demonstration that it requires thousands of times as much antitoxin to cure an animal, after the symptoms have developed, as it does to prevent the development of the disease by giving antitoxin soon after the animal is infected ; hence the necessity of prophylactic injections in cases of suspicious wounds. This serum is dispensed as a fluid or in a dry form.

*Antitubercle Serum.*—This serum is prepared in the usual way from the blood of horses that have been immunised against attenuated cultures of the tubercle germ or their poisonous product—old tuberculin. Considerable claims for this serum as a curative agent have been made. About a year ago Koch devised a mechanical process of breaking the wax-like enveloping membrane of the tubercle bacilli and obtaining the contained constituents, which when properly prepared he called tuberculin T. R. Buchner about the same time originated a similar method of extraction. Recently antitubercle serums have been placed on the market which were obtained from the blood of animals immunised against this material, but the results obtained give little promise of success.

*Antistreptococcic Serum.*—It is now generally believed that erysipelas, puerperal fever and many septicemias are due to streptococci of the same or closely related species, and that these germs often attack the weakened tissues and complicate such diseases as scarlatina, diphtheria tuberculosis and the like. It was early perceived that a serum efficacious against these germs, or their toxins, would have a wide field of usefulness. Marmorek first succeeded in producing such a serum, which is now prepared in this country and is being quite extensively employed. While we cannot yet come to a positive decision regarding its merits, the results following its administration in cases of puerperal fever, erysipelas, peritonitis and other forms of septic poisoning are, indeed, very encouraging.

Antityphoid, antibubonic, antisyphilitic, antirabic and other serums have been prepared. I believe we should regard all the antitoxins on the market at the present time, with the exception of antidiphtheritic serum, and possibly antistreptococcic serum, as experimental remedies which may or may not produce the results desired, but fortunately they can be employed with other therapeutic agents and they do no harm at most.

We find, briefly, that immunity can be conferred against a great variety of substances of animal, vegetable and bacterial origin. This immunity may be produced by feeding the poison, or by injecting it subcutaneously or intravenously; it can be taken from one animal and administered to another to aid the tissues in their efforts to prevent the entrance of disease organisms, or to destroy such organisms after they have gained access to the citadel of life. The immunising substance is found to be present in varying quantities in the different fluids and organs of the animal body, but at present we are unable to say in which this protecting substance is formed. Much of the recent work shows that the white corpuscles have a great deal to do with its production.

The great success of serum therapy in the treatment of diphtheria justifies the conclusion that rational medicine may now justly claim to be one of the most progressive of the sciences.

